# Giant extragastrointestinal stromal tumor in the transverse mesocolon concomitant with gastric cancer in an elderly patient: Case report

**DOI:** 10.3892/ol.2012.1030

**Published:** 2012-11-16

**Authors:** DONG XUE, HONGQIANG CHEN, YUXIN CHEN

**Affiliations:** Department of General Surgery, Qilu Hospital, Shandong University, Jinan 250012, P.R. China

**Keywords:** extragastrointestinal stromal tumors, gastric cancer, transverse mesocolon

## Abstract

Extragastrointestinal stromal tumors (EGISTs) are neoplasms located outside the gastrointestinal tract in sites including the omentum, mesentery and retroperitoneum. EGISTs of the transverse mesocolon are rarely noted in the literature. Herein, we describe a rare case of giant EGIST concomitant with gastric cancer in a 78-year-old male who presented with upper abdominal pain and a palpable mass. The patient underwent en bloc resection of the tumor with a distal gastrectomy, with a D2 lymphadenectomy for the gastric cancer, accompanied with resection of a segment of the transverse colon. The patient received targeted therapy (imatinib 400 mg, daily) and adjuvant chemotherapy with FOLFOX (six cycles). Neither recurrence nor metastasis was observed after 24 months of follow-up.

## Introduction

Gastrointestinal stromal tumors (GISTs) are rare neoplasms that arise from mesenchymal cells of the gastrointestinal tract. Extragastrointestinal stromal tumors (EGISTs) have no contact with the stomach and intestine, by definition, and typically occur in the omentum, mesentery or retroperitoneum (1,2). However, EGISTs are not connected to the digestive tract (3). GISTs in the omentum or mesentery are typically metastases from primary sites within the gastrointestinal tract. EGISTs are often included in large studies of stromal tumors, in which they account for less than 10% of overall cases. EGISTs have rarely been identified in the pancreas, diaphragm, spleen, pelvis or abdominal wall (4–7).

The most common type of carcinoma associated with GISTs is gastric carcinoma (8). To the best of our knowledge, primary EGIST concomitant with digestive tract carcinoma is rarely encountered. We describe a rare case of giant EGIST in the transverse mesocolon concomitant with gastric adenocarcinoma, in a 78-year-old male who presented with upper abdominal pain and a palpable mass. We discuss the specific recommended therapies for patients with concomitant EGST and gastric cancer, with a review of the literature.

The study was approved by the Ethics Committee of Qilu Hospital, Shandong University, China. The patient’s family consented to this study.

## Case report

**Figure 1.**
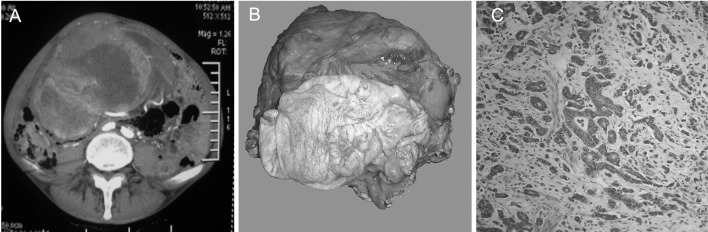
(A) Abdominal computed tomography (CT) scan showing a heterogenous mass occupying most of the middle and lower abdomen. (B) The mass, part of the transverse colon and the distal stomach were resected en bloc. (C) Pathologic sections of the gastric adenocarcinoma with poor to moderate differentiation (hematoxylin and eosin stain, x200).

A 78-year-old male was admitted to hospital for abdominal distension and pain. The patient had no history of epigastralgia or peptic ulcers and there was no significant relevant family history. A physical examination revealed a 20x12 cm palpable mass in the middle and lower abdomen, with minimal intrinsic mobility. Carcinoembryonic antigen (CEA), cancer antigen (CA) 724 and carbohydrate antigen (CA) 19-9 levels were normal. A computed tomography (CT) scan revealed a 21x14 cm heterogeneous mass extending from the fundus to the mid-abdomen, with a number of solid and cystic components within the tumor (Fig. 1A). A gastroscopy revealed an intraluminal ulcer in the lower posterior wall of the gastric antrum; biopsy specimens were obtained. No metastatic lesions were identified in any other organs in either the abdominal ultrasonography or the CT scan. A biopsy confirmed the presence of an adenocarcinoma of the stomach with poor to moderate differentiation. A laparotomy revealed an extremely large tumor (22x15 cm) arising from the transverse mesocolon and adjacent to the greater curvature of the stomach. The mass was in close apposition to the pancreas and duodenum. There was no evidence of invasion or underlying peritoneal infiltration. The mass was resected en bloc with part of the transverse colon, and distal gastric resection was performed (Fig. 1B).

**Figure 2.**
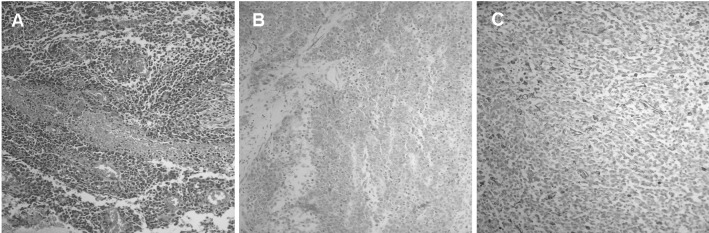
(A) The tumor is mostly composed of epithelioid cells with focal necrosis, fibrosis and hemorrhagic areas (hematoxylin and eosin staining, x200). (B) Immunohistochemically, the epithelioid cells demonstrated cytoplasmic staining for CD117 (c-kit) (x200). (C) Immunohistochemically, the epithelioid cells demonstrated cytoplasmic staining for desmin (x200).

Macroscopic examination of the distal gastrectomy specimen revealed a Borrmann type II tumor in the stomach, measuring 3x3 cm. On histopathological examination, the tumor was identified to be an adenocarcinoma with poor to moderate differentiation that exhibited transmural infiltration (Fig. 1C). No nerve or vascular invasion was evident. Two lymph node metastases were detected in 28 retrieved lymph nodes. According to the TNM classification, the gastric carcinoma was stage IIIA. Histopathological examination demonstrated that the extremely large mass was mainly comprised of epithelioid cells, and revealed focal necrosis, fibrosis and hemorrhagic areas (Fig. 2A). Mitotic figures were recognized in 15 out of 50 high-power fields. Immunohistochemical analysis revealed that CD117 (Fig. 2B) and desmin (Fig. 2C) were positive in the neoplastic cells. However, tumor cells exhibited negative expression for CD34, S-100 and smooth muscle actin (SMA). Based on these results, the diagnosis was an EGIST of the transverse mesocolon that had not originated from the digestive tract. The postoperative period of the patient was uneventful; the patient was discharged two weeks after surgery. The patient was started on adjuvant chemotherapy with a FOLFOX regimen: oxaliplatin IV, 85 mg/m^2^ (on day 1); leucovorin IV, 200 mg/m^2^ (on days 1 and 2); 5-FU (fluorouracil) IV, 400 mg/m^2^ (on days 1 and 2) and 5-FU IV 22 hours in fusion, 600 mg/m^2^ (on days 1 and 2). This regimen was repeated every 2 weeks for 6 cycles. Additionally, the patient was simultaneously administered imatinib at a dose of 400 mg/day for 1 year. No evidence of tumor recurrence was identified after 24 months of follow-up.

## Discussion

GISTs identified outside the gastrointestinal tract as apparent primary tumors are designated as EGISTs. EGISTs may occur in the omentum, mesentery or retroperitoneum, adjacent to (although separate from) the stomach and intestine. Their true origin is uncertain; however, their histological appearance and immunophenotypes are typically identical to those of classical GISTs. The EGIST in the present case was located inside the mesocolon, outside the gastrointestinal tract. EGISTs of the mesocolon have rarely been noted in the literature (9–11). The pathogenesis of EGIST concomitant with abdominal malignancy is still unknown. Only sporadic cases have been described. EGISTs of the greater omentum (10 and 8 mm in diameter, respectively) have been identified by coincidence when a gastrectomy was performed (12). EGISTs are often asymptomatic as they lack mucosal participation, whereas GISTs commonly present with gastrointestinal or intratumoral bleeding (13,14). Due to their their anatomic site, EGISTs typically grow larger than GISTs and only present clinical symptoms after a significant period of time (15,16). Certain studies have described gastric stromal tumors to be synchronous with gastric malignancy. To the best of our knowledge, a giant EGIST in the transverse mesocolon concomitant with gastric carcinoma has never been studied.

The clinicopathological or immunohistochemical features of EGISTs and GISTs were not observed to be markedly different. Primary EGISTs of the omentum and mesentery demonstrated clinicopathological and immunohistochemical characteristics similar to a GIST of the digestive tract described previously in the literature (17). This supports the hypothesis that these tumors originate from extragastrointestinal c-kit positive cells. Their histogenesis, criteria for diagnosis, prognostic factors and classification have been debatable and controversial. GISTs are accurately diagnosed by their morphology and immunophenotyping. Histologically, there are three types of GISTs: spindle, epithelioid and mixed. The diagnosis of an EGIST in the present case was in accordance with the histological and immunohistochemical criteria; the tumor was epithelioid-type, and was immunopositive for CD117 and desmin. More than 95% of EGISTs express CD117, the c-kit proto-oncogene protein that is a transmembrane receptor for the stem cell growth factor; while 50–100% express CD34, the human progenitor cell antigen. It is less common for EGISTs to stain positively for SMA, S-100 and desmin (6,17,18). Zheng *et al* demonstrated that the c-kit and PDGFRA mutation in EGISTs was similar to that of GISTs. Therefore, from a molecular genetics perspective, EGISTs may be a unique subtype of GISTs (3).

The risk categories of EGISTs are contested. A tumor size >10 cm, mitotic activity >2 per 50 high-power fields, tumor necrosis, marked nuclear atypia, >10% Ki-67 protein expression and epithelioid-/mixed cell-type, have acted as significant predictors of survival (2,3). Survival analysis indicated that mitotic count and Ki-67 protein expression levels were significant predictors of disease-specific survival; however, tumor size, primary location, c-kit and PDGFRA gene mutations were not. The high risk of recurrence and prognostic factors for survival are controversial. This may be due to a limited number of samples, a different composition of ages and different histologic judgements of pathologists across studies. Therefore, it is necessary to enlarge the sample size in order to reach a precise conclusion. EGISTs are capable of remaining clinically silent, irrespective of their large size, which exceeded 10 cm in the majority of published cases. EGISTs are rarely encountered due to the fact that they seldom produce symptoms that would lead to their detection. Tumor size has not been observed to be a reliable prognostic parameter in EGISTs. In the present case, focal necrosis, >10% Ki-67 protein expression and a high-power mitotic count >10 resulted in the tumor being considered high-risk. The tumor in the present case demonstrated epithelioid-type histology. Positive immunohistochemical staining for CD117 is also a defining feature of EGISTs, and it correlates with a tumor response to treatment with the KIT kinase activity inhibitor. The present case demonstrated strong positive staining for CD117 and desmin, as well as negative staining for CD34, S-100 and SMA, by immunohistochemistry. We propose that tumor coexistence is a unique event in histology.

There are few data regarding the clinicopathological factors of EGISTs that predict the patient’s prognosis. In a study by Reith *et al*, 39% of patients with EGISTs had an adverse outcome, which suggested that EGISTs were aggressive and were more similar to GISTs located in the distal gastrointestinal tract (2). Barros *et al* analyzed 9 EGISTs and revealed that the average overall survival was 26.4 months (6). Llenas-García *et al* demonstrated that 66.6% of mesenteric cases with a high mitotic rate exibited hepatic metastasis at 6 and 32 months, respectively (17). It was suggested that EGISTs that originated from a mesenteric location had an aggressive course that was more similar to that of small intestinal, as opposed to gastric stromal tumors.

Surgery is the standard treatment for non-metastatic EGISTs. A preoperative diagnosis is difficult, and the patient undergoes an operation based on the diagnosis of an abdominal mass. Where possible, en bloc resection with contiguous tissues and regional lymph nodes is conducted. It is crucial for the pathologist to examine whether the tumor is adhesive to the gastrointestinal wall or other tissues. A histological diagnosis of EGIST is often unsatisfactory. In the present case, the patient underwent an en bloc resection of the tumor and partial transverse mesocolon, accompanied with a distal gastrectomy with a D2 lymphadenectomy. Positive immunohistochemical staining for CD117 is a defining feature of EGISTs. C-kit-positive tumors are most responsive to treatment with a c-kit selective tyrosine kinase inhibitor. Due to the advent of targeted therapy, imatinib, an inhibitor of the tyrosine kinase activity of c-kit, has revolutionized the treatment of this disease. The recommended first-line treatment of advanced GIST is 400 mg/day imatinib (19). In certain patients, an escalated dose (800 mg/day) has achieved tumor control and conferred further survival benefits after failure with 400 mg/day (20). Adjuvant chemotherapy (FOLFOX) and imatinib were applied in the present case. The patient was followed up and was free of recurrence 24 months after surgery.

The coexistence of EGIST with other abdominal malignancies is a rare phenomenon; we suggest that their synchronous occurrence may be a coincidence. Although there are data regarding the co-occurrence of EGISTs and other malignant tumors, the mechanism of synchronous tumorigenesis remains unclear. Surgical resection is currently the mainstay of treatment for EGISTs. The existing data on the coexistence of EGISTs with other types of gastric carcinoma are insufficient to reach a final conclusion regarding the treatment, prognosis and recurrence.

In summary, the present case was a giant EGIST in the mesocolon accompanied with gastric cancer in an elderly male. We stress that, although their prevalence is very low, surgery-based multimodal treatment is the preferred strategy for compound tumors. Adjuvant chemotherapy and targeted therapy are important for high-risk EGISTs accompanied with digestive tract malignancies. Due to the limited number of cases, the existence of a common tumorigenesis factor among the different tumors cannot be identified. Further studies are required to expound the possible association.
